# Molecular genetics of neuropsychiatric illness: some musings

**DOI:** 10.3389/fgene.2023.1203017

**Published:** 2023-11-01

**Authors:** Meghana Janardhanan, Somdatta Sen, Bhagylakshmi Shankarappa, Meera Purushottam

**Affiliations:** Molecular Genetics Laboratory, Department of Psychiatry, National Institute of Mental Health and Neurosciences, Bengaluru, India

**Keywords:** *APOE ε4*, *TOMM40*, *PLA2G6*, genetic risk score, severe mental illness, cirrhosis

## Abstract

Research into the genetic underpinnings of neuropsychiatric illness has occurred at many levels. As more information accumulates, it appears that many approaches may each offer their unique perspective. The search for low penetrance and common variants, that may mediate risk, has necessitated the formation of many international consortia, to pool resources, and achieve the large sample sizes needed to discover these variants. There has been the parallel development of statistical methods to analyse large datasets and present summary statistics which allows data comparison across studies. Even so, the results of studies on well-characterised clinical datasets of modest sizes can be enlightening and provide important clues to understanding these complex disorders. We describe the use of common variants, at multiallelic loci like *TOMM40* and *APOE* to study dementia, weighted genetic risk scores for alcohol-induced liver cirrhosis and whole exome sequencing to identify rare variants in genes like *PLA2G6* in familial psychoses and schizophrenia in our Indian population.

## Graphical Abstract

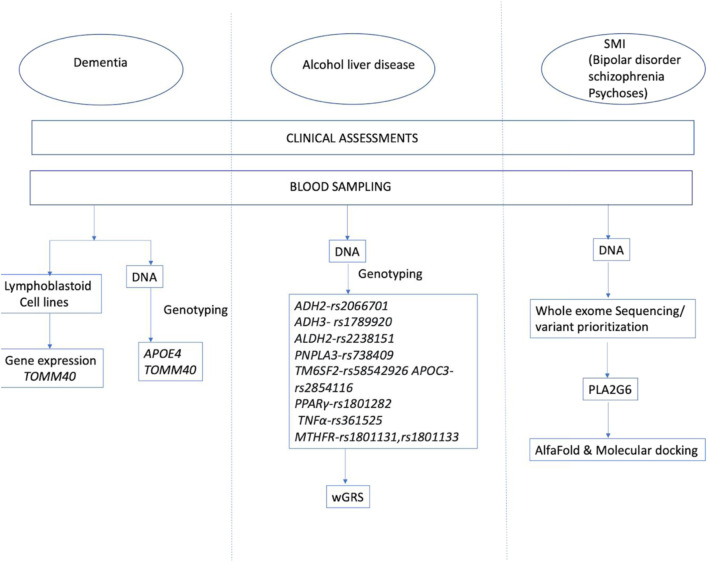

## Introduction

The idea that many forms of mental illness are inherited has always been suspected, but formal methods to assess this emerged only some decades ago. Follow-up analysis of twins, reared separately and away from parents, who themselves had a diagnosis of a psychiatric illness, seemed to suggest that the susceptibility to psychiatric illness in adulthood might be already encoded in the genetics (Gottesman and Shields, 1976). These are “common” syndromes and the search for these genetic factors has had many twists and turns. We will need a more comprehensive understanding of genetic variations and biology to translate these advances into a better understanding of disease.

### The application of classical genetics methods

The early studies of disorders relied upon the methods of “classical” Mendelian laws and linkage, wherein an attempt was made to identify the segment of the genome that was shared by ill persons within an affected family. This strategy worked best when the disease was highly penetrant, was inherited in a dominant or recessive manner, and there were large multi-generation families with multiple meioses that could be analysed (Friedman et al., 2021). A crucial part of this analysis was the affectation status and accurate phenotyping. From the early analysis of large pedigrees from Iceland, to the discovery of a translocation in Chr1:11 that included the *DISC1* gene as segregating with risk of psychoses, a number of studies with large family structures explored the genetic risk of psychiatric illness, with complex, and often confounding results (Sherrington et al., 1988; Millar et al., 2000; Craddock et al., 2005). We used linkage analysis, supplemented with a family-based association test (Transmission disequilibrium test), to detect evidence of shared liability in more than 50 families having multiple affected members. We observed a positive linkage and association finding at 18p11.2 for psychosis (Mukherjee et al., 2006). Gradually as more reports came in worldwide, linkage hits appeared on many chromosomal regions for psychiatric illness, though these were often not reproducible across studies, and it became apparent that there could be private mutations in particular clinical populations (Xu et al., 2009). The use of endophenotypes to be able to describe a complex disorder was thought necessary (Braff et al., 2007). However, even in pedigrees with many affected individuals, there was a lot of variation in illness presentation. The varying penetrance and heterogeneity in what was otherwise a relatively common disease led to the evolution of the common disease, common variant hypothesis (Mukherjee et al., 2002).

The *DISC1* locus was first discovered as part of a chromosomal t (1; 11) (q42.1; q14.3) translocation that seemed to add to risk of both schizophrenia and bipolar disorder, in a large Scottish family (St Clair et al., 1990). This was later evaluated for association with schizophrenia by genotyping Single Nucleotide Polymorphisms (SNPs), and also sequencing by a large number of groups (Ma et al., 2018), but with conflicting results. A targeted genotyping of the SNPs in the gene resulted in only a partial gender-specific association in our sample set of 1,252 individuals (419 bipolar disorder patients, 408 schizophrenia patients and 425 controls) (Murthy et al., 2012). As large-scale genotyping methods for common variants became possible, this locus did not retain its significance across other populations, perhaps hinting again at the heterogeneity of mental illness, and its patterns of inheritance (Mathieson et al., 2012).

One interesting outcome of the Genome-wide association (GWA) method was the feasibility of comparing genotype results for multiple loci across populations and studies. This showed that the variation in population allele frequencies might affect the detection of association, across populations (Asif et al., 2021).

### Dementia and APOE Ɛ4

The experience with the *APOE* Ɛ4 haplotype and risk of dementia is thus exemplary. The *APOE* Ɛ4 haplotype, which is a combination of two missense variants, was first described by the Roses lab at the Duke University Medical Centre as a causal locus for dementia (Corder et al., 1993), and has been associated with dementia in populations across the world, though with varying effect sizes. Our studies confirmed that the frequency of the risk allele was much higher in those with dementia (20%) when compared to age-matched controls (12%) i.e., the Apoe4 carrier allele frequency was 14.8% in controls and 33% in dementia patients (Bharath et al., 2010). The global reported average proportion of APOE4 carriers is 23.9%, and varies with geography (from 19.3% to 30.0%), and ethnicity (from 19.1% to 37.5%) (Wang et al., 2021).

The intersection between variations in frequency of the *APOE* Ɛ4 alleles, and the prevalence of dementia thus becomes important. A recent examination of *APOE* Ɛ4 carrier frequency and dementia prevalence by Llibre-Guerra et al. (2023) showed a similar *APOE* Ɛ4 carrier frequency in Caribbean and American Hispanics of about 21%–23%, with a dementia prevalence of 9%–10% in these populations. Interestingly while the carrier frequencies for the non-Hispanic whites and African Americans are 25% and 34%, the prevalence of dementia is seen to be 3.2% and 13.3% for these two populations. The estimated dementia prevalence for adults older than 60 in India is 7.4%, with significant age and education gradients, sex and urban/rural differences, and cross-state variation (Lee et al., 2023). These differences could be due to both additional risk factors or other environmental and dietary factors.

We have attempted to identify other modifying variants related to the risk of dementia. The *CLU* (Clusterin/ApoJ)—rs11136000) and *PICALM* (phosphatidylinositol binding clathrin assembly protein)—rs3851179) loci were reported in a two-stage genome-wide association study of AD involving over 16,000 individuals from Europe and the United States (Harold et al., 2009). These results were not replicated in our modest sample of AD cases and age-matched controls; we observed an MAF of 0.29 for *CLU* (rs11136000) and 0.43 for *PICALM* (rs3851179) in controls (Shankarappa et al., 2017). We were soon joined by the other groups that studied the East Asian, Turkish, Polish and African American populations who also did not find an association at these loci (Logue et al., 2011; Klimkowicz-Mrowiec et al., 2013; Sen et al., 2015; Han et al., 2018). There appeared to be a particular allele frequency that was showing association, especially in the case of the *CLU* locus. It was concluded that perhaps the same variant might not show an association but a larger haplotype analysis might be useful to determine whether the gene locus was important in Alzheimer’s disease.

Analysis of the region around the *APOE* Ɛ4 locus identified a polymorphism in the *TOMM40* gene which codes for a protein in the outer mitochondrial membrane (Roses et al., 2010). The variation in the length of the polyT tract in the sixth intron of this gene (resulting in alleles S, L, VL) showed a significant association with dementia (Gottschalk et al., 2014). While the *TOMM40* gene biology suggests that it could contribute independently to the risk of dementia, its proximity to the *APOE* gene could lead to a spurious association in genetic analyses. The results have varied across populations depending upon the linkage disequilibrium between the *TOMM40* locus (rs10524523) and the *APOE* locus. In the European population, 0.8% of the *APOE* Ɛ4 (−) and 94.2% of *APOE* Ɛ4 (+) had the ‘L’ allele. In the African-American population, 1.1% of the *APOE* Ɛ4 (−) had L, but only 47.8% of the *APOE* Ɛ4 (+) had the L allele (Yu et al., 2007). We thus genotyped the TOMM40 locus in our dementia population along with the *APOE* Ɛ4 status.

### Effect of environment

Populations may differ in their genetic predisposition to damage caused by environmental factors. Alcohol related disease may thus be viewed as a particular natural experiment, wherein the risk of disease is contingent upon exposure. The two major enzymes involved in alcohol metabolism show significant ethnic variation. Alcohol dehydrogenase (ADH), converts alcohol to acetaldehyde, which is then converted to acetate by aldehyde dehydrogenase (ALDH). Seven known *ADH* genes encode enzymes that catalyze the conversion of alcohol to aldehyde (Osier et al., 2002). However, the relative kinetics of the enzyme variants in an individual might dictate the ability to use large quantities of alcohol and not feel uncomfortable. This could add to the risk of disease, as a feeling of discomfort would discourage excessive drinking, and perhaps be protective. Differences in the frequency of variants in the *ADH* and *ALDH* genes contribute to the “flushing” response in East Asians, and they are thought to be more sensitive to the effects of alcohol, as compared to Europeans. Recent reports from East Asia have linked the *ALDH2* allele to flushing and discomfort on alcohol consumption (Lee et al., 2014). Interestingly, Indians were thought to be more sensitive to alcohol, as compared to Europeans, in early Greek writings from 1,500 years ago (Lucian of Samosata, 2018). A study in our population, however, could not detect the ALDH2 isoform associated with increased aldehyde accumulation as observed in East Asians. The protective allele may thus be rare in the Indian population. These differences in the oxidative pathways could also impact other metabolic processes, and lead to adverse consequences on the liver (Li et al., 2015). Most patients who use alcohol develop liver steatosis over time, although further progression to cirrhosis is not universal. A reliable prediction of susceptibility to liver complications from alcohol abuse would thus be useful.

### Whole exome sequencing studies

The wider availability of genomic screens now allows us to interrogate families with multiple affected members through other methods. Whole exome sequences (WES) and whole genome sequences can provide additional insight, especially on a case-by-case, or even a familial basis (Kato et al., 2023). The exome constitutes less than 3% of the genome, but since it directly impacts protein function, variants may have a much larger impact on biological processes, and thus susceptibility to disease. Such a penetrant coding variant may be sufficient in itself, or interact with a background of common variants (Kato et al., 2023).

About 10% of dementia patients have another first-degree relative with dementia (Reitz et al., 2023). We did a WES study, on a set of such individuals, and were able to identify several rare deleterious genetic variations, in the coding region of genes involved in amyloid signalling (*PSEN1, PLAT* and *SORL1*) and other dementia-associated pathways (Syama et al., 2018). We detected a different base change for a previously reported mutation in the *PSEN1* gene (TGG to TGT/C) resulting in an identical W165C missense mutation, highlighting the possibility of different DNA variants across families or populations causing changes in the same risk regions of candidate genes.

**FIGURE 1:**
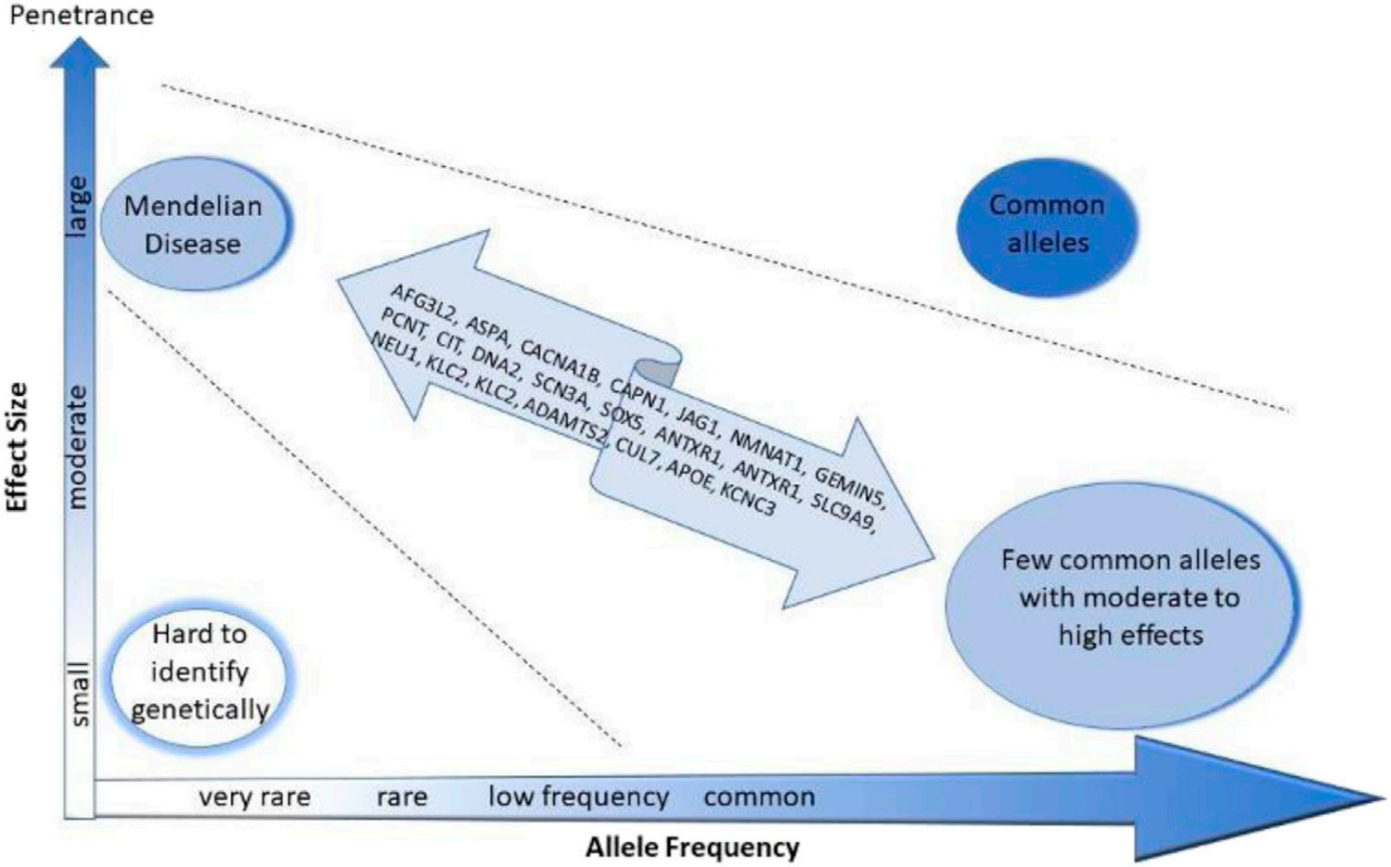
The putative risk loci identified by whole exome sequencing of familial mental illness might be low penetrance risk variants.

We also used WES in a set of families with multiple affected members with schizophrenia, bipolar disorder, dementia and OCD and identified many plausible risk variants in familial severe mental illness (SMI) (Figure 1) (Ganesh et al., 2022). We detect potentially deleterious variants in genes and pathways that have been implicated in Mendelian disorders, and also previous GWA studies (Ganesh et al., 2022). Interestingly some of these individuals also shared some of the clinical characteristics of the same Mendelian disorders (unpublished observations). Thus, many risk alleles for genetic diseases seem to produce a spectrum of syndromes, depending on whether they occur as heterozygous, homozygous or compound heterozygotes.

This possible intersection with the distribution of variants, across populations and within a gene and protein at particular points of vulnerability, is exemplified by our observations on the *PLA2G6* gene. The Phospholipase A2 Group 6 (*PLA2G6*) gene located on chromosome 22q13.11 consists of 17 exons and encodes 806-amino acid VIA calcium-independent phospholipase A2 protein (Chu et al., 2020). Homozygous missense variants (e.g., p.D331Y, p.R741Q), are often linked to atypical INAD (infantile neuroaxonal dystrophy) and early onset levodopa-responsive parkinsonism, with variable brain iron accumulation (Roeben et al., 2023). Many reports also found neuropsychiatric symptoms or behavioral changes in the initial presentations of patients with *PLA2G6* mutations (Chu et al., 2020). Abnormalities in phospholipid biosynthesis, membrane remodelling and related processes have been implicated in many neuropsychiatric syndromes (Minghui et al., 2022). Increased phospholipase A2 (PLA2) activity has been reported in schizophrenia, and treatment with antipsychotic drugs reduced the enzyme activity to levels similar to those in control (Gattaz et al., 1987). Thus, it was crucial to study variants of *PLA2G6* in SMI.

The studies on the molecular genetics of neuropsychiatric illness now have to encompass a wide spectrum, from issues of penetrance to the range of minor allele frequencies. This may require a reconsideration of many of the prevailing models of classifying and understanding the impact of genetic variations, as recently discussed by Yao et al. (2023). We describe a few encounters with these phenomena in our work (summarised in Abstract Figure), which may have made this quest seem worthwhile.

## Methods

### Dementia studies

#### Clinical assessments

Patients diagnosed with AD, and age-matched controls (Sadanand et al., 2013), were identified from the Geriatric Clinic service at the National Institute of Mental Health And Neurosciences (NIMHANS), India after informed consent. The patients underwent cognitive assessment, using standard instruments; Hindi Mental State Examination (HMSE) (Ganguli et al., 1995), Everyday Abilities Scale for India (EASI) (Fillenbaum et al., 1999) and Clinical dementia rating (CDR) (Morris, 1993). The sample demographics are summarised in Supplementary Table S1. Ten millilitres of whole blood was collected in EDTA tubes. DNA was isolated using Miller’s salting out method (Miller et al., 1988).

#### Genotyping at APOE and TOMM40

*APOE* genotypes were determined by Amplification-Refractory Mutation System Polymerase Chain Reaction (ARMS PCR) (Pantelidis et al., 2003). A subset of samples was confirmed for their *APOE* status by Sanger’s sequencing. Poly T repeat of *TOMM40* was analysed by PCR followed by fragment analysis in 311 samples (dementia (N = 151) and age-matched controls (N = 165)) on ABI 3500XL Genetic Analyser (Linnertz et al., 2012).

#### Gene expression

Lymphoblastoid cell lines (LCLs) were generated from lymphocytes of AD patients (N = 29) and age-matched controls (N = 16) by Epstein–Barr virus (EBV) transformation (Neitzel, 1986). For the gene expression experiment, 3 million cells were taken at passage 8. RNA was isolated using the Trizol extraction method. The quality and integrity of RNA was checked before conversion to cDNA. *UBC* was chosen as the housekeeping gene. Expression was represented as the expression of the gene relative to the housekeeping gene calculated by 2^−ΔΔCT^.

#### Statistical analysis

Hardy Weinberg equilibrium did not deviate in cases and controls for both genes. Chi-square test was used for association studies. The power of the study is >95% for the association study. For expression studies, normality was calculated using the Shapiro-Wilk test and appropriate (independent T/Mann Whitney) tests were used to compare the difference in mean between the groups.

### Alcohol-induced liver cirrhosis

#### Clinical assessments

Men with Alcohol Use Disorder (AUD) with Cirrhosis (AUDC + ve, N = 131) and AUD without Cirrhosis (AUDC-ve, N = 105) based on International Classification of Mental and Behavioural Disorders (ICD) 10 criteria, identified from the clinical services of St John’s Medical College Hospital (Gastroenterology and Psychiatry), Bengaluru, India; and the Centre for Addiction Medicine at NIMHANS, participated in the study. Fibroscan and/or sonographic findings were used to rule out fibrosis (Fib-4 <3.25, Liver Stiffness Measurement, LSM <14kPa/) in the AUDC-ve group.

#### Genetic risk score calculation

A genetic risk score (GRS) is an estimate of the cumulative contribution of genetic factors to a specific outcome of interest in an individual. The score may take into account the reported effect sizes for those alleles and may be normalized by adjusting for the total number of risk alleles and effect sizes evaluated (Igo et al., 2019). South Asian (SAS) (N = 260) 1KGP data was used to calculate Genetic Risk Score (GRS) in the population. A total of 10 SNPs, from genes involved in alcohol metabolism (*ADH2* - rs2066701, *ADH3*-rs1789920, *ALDH2*-rs2238151); lipid metabolism (*PNPLA3*-rs738409, *TM6SF2*-rs58542926, *APOC3*-rs2854116); cytokine (*PPARγ*-rs1801282, *TNFα*-rs361525) and one-carbon metabolism (*MTHFR*-rs1801131 and rs1801133) known to be associated with the risk of AUDC + ve (Dutta, 2013; Roy et al., 2016), were assessed in this study. Genotypes were generated for the SNPs using PCR-RFLP (Polymerase Chain Reaction- Restriction Fragment Length Polymorphism). GWAS and association studies have shown that these SNPs are associated with alcohol use disorder with cirrhosis (Fabris et al., 2009; Dutta, 2013; Singh et al., 2014; Buch et al., 2015). Population standardized weighted genetic risk scores (wGRS) were calculated based on the effect size from the PGS catalogue for alcoholic cirrhosis (PGS Catalog - PGS000704)

$$\text{wGRS}=\frac{\left[\beta 1\left(\text{risk}\;\text{allele}\;\text{count}\;\text{of}\;\text{SNP}1\right)+\beta 2\left(\text{risk}\;\text{allele}\;\text{count}\;\text{of}\;\text{SNP}2\right)+\ldots ..\beta n\left(\text{risk}\;\text{allele}\;\text{count}\;\text{of}\;\text{SNPn}\right)\right]}{\text{Sum}\;\text{of}\;\text{the}\;\beta }*\text{numberof risk loci}$$

### Whole exome sequences of severe mental illness

#### Screening for PLA2G6 variants

In order to check for *PLA2G6* variants in the available exome data of families with severe mental illness (SMI) we examined WES data of 310 exomes, (Ganesh et al., 2022), which included those with SMI (n = 190) and controls (familial n = 60; population n = 60). To discriminate the impact of a variant as disease-causing or neutral, we employed various *in-silico* prediction tools such as SIFT, LRT, MutationTaster, FATHMM and MetaSVM from Varsome (Kopanos et al., 2019). *In-silico* prediction methods and molecular docking analyses were used to understand the consequence of the variants in the *PLA2G6* mutations that were identified.

#### Molecular docking analysis

We carried out molecular docking analysis of antipsychotic drugs with the predicted AlphaFold structure of the protein to evaluate the effect of these variants on drug binding. The full-length predicted protein structure of human PLA2G6 was imported to the Schrodinger Maestro software package (Maestro, Schrödinger, LLC) and the protein was prepared by adding hydrogen atoms and assigning proper bond orders. Prime (Jacobson et al., 2004) was used to fill in missing side chains, and Epik was used to generate protonation states with a pH of 7.0±2.0 (Shelley et al., 2007). The protein structure was further optimized by PRCG (Polak-Ribier Conjugate Gradient) (Polak and Ribiere, 1969) minimization method with a maximum of 2,500 iterations and converge threshold of 0.05. The total energy of the system after minimization was −168465.094 kJ/mol. SAVES v6.0 server (https://saves.mbi.ucla.edu/) was used to assess the quality of the model. Results from the Ramachandran plot showed 90.1% and 9.1% of the residues to be in the most favoured regions and additional allowed regions.

The possible drug-binding sites were identified using the SiteMap method (Halgren, 2009). Three potential binding sites (site1, site2 and site3) with high druggability scores (DScore >1) have been identified in the catalytic domain of the receptor including the interface region. Prior to docking, all the hydrogen atoms were removed from the protein and only the polar hydrogens were added and Gasteiger Charges were computed. The grid box was centred in the catalytic region of the receptor (X: −15.188 Y: 7.689 Z: 17.443) and the number of grid points in XYZ directions was set to 56*40*40 with a grid spacing of 0.909 Å, such that the grid box covered the whole catalytic-site as well as the interface region between the catalytic and ankyrin repeat domains. The docking calculations were performed using AutoDockTools (ADT) v1.5.6 (Morris, 1993) and AutoDock Vina (Trott, O. et al., 2010) with default settings.

## Results

We describe here the work carried out by our group, for three different syndromes, highlighting the importance of a multipronged approach to better understand genetic correlates of complex neuropsychiatric disorders.

### Genetics of dementia

At NIMHANS, our sample set of 464 unaffected adults older than 60, had an *APOE* Ɛ4 carrier allele frequency of 18.3%. On genotyping the *TOMM40* poly A locus in our unaffected elderly population (N = 165) we found, 3.6% of *APOE* Ɛ4 (−) and 37% of *APOE* Ɛ4 (+) had the “L” allele. As shown in Table 1, a comparison of individuals with dementia (N = 151) and age-matched controls (N = 165) showed a significantly higher occurrence of the “L” allele with *APOE* Ɛ4 carriers and dementia (*p* < 0.0001). Further, even among the *APOE* Ɛ4 non-carriers, the “L” allele frequency was significantly higher in cases than in controls (*p* < 0.0008) showing that the L allele on its own may also be a risk factor for dementia in our sample set. We also checked for LD across these two genes with the 3 markers (rs10524523, rs429358, rs7412) in our population. The linkage between the *TOMM40* locus and *APOE* though present, appeared to be much weaker compared to that reported in European populations (Yu et al., 2007).

**TABLE 1:** Distribution of TOMM40 alleles in AD cases and controls based on APOE Ɛ4 carrier status.

		S n (%)	L n (%)	VL (n) %	p value^a^
*APOE* Ɛ4 non-carriers	Cases	71 (40)	23 (13)	82 (47)	*p* < 0.0008
	Controls	129 (46.7)	10 (3.6)	137 (49.6)	
*APOE* Ɛ4 carriers	Cases	34 (27)	58 (46)	34 (27)	*p* < 0.0001
	Controls	18 (33)	20 (37)	16 (30)	

In both APOE Ɛ4 carriers and non carriers, the ‘L’ allele frequency is significantly higher in cases compared to controls.

We hypothesised that gene expression of *TOMM40* could be influenced by the genotype at this intron 6 locus and carried out gene expression studies. However, in a study of 45 LCLs, two-thirds of which were derived from AD patients, there was no influence of the *TOMM40* allele on gene expression. The *APOE* Ɛ4 carrier status also did not have any effect on *TOMM40* gene expression in LCLs (Supplementary Figure S1). It is, however, possible that there may be tissue-specific effects of this polymorphism which are not seen in LCLs.

### Alcohol dependence genetics

We compared individuals who had developed cirrhosis (AUDC+), to others who had not (AUDC-ve); though both had been using alcohol. The demographic and clinical characteristics of participants (N = 236) showed that the two groups did not differ in age, but those in the AUDC + ve group had used slightly lesser units of alcohol (13 ± 6) compared to the AUDC-ve group (16 ± 7) (*p* = 0.01) (Supplementary Table S2). The AUDC + ve group had also been drinking for a comparatively shorter duration (16 ± 7 years vs. 18 ± 8 years) and had a later age of onset of drinking (29 ± 8 years vs. 23 ± 7 years) (*p* = <0.001). AUDC + ve group showed more evidence of liver disease, in the form of higher concentrations of serum total and direct bilirubin, Alkaline Phosphatase (ALP), Gamma- Glutamyl Transferase (GGT) and lower concentrations of total protein, albumin, and haemoglobin levels, when compared to AUDC-ve group.

In order to check for a genetic predisposition if any, for adverse consequences of alcohol exposure we computed the genetic risk score (GRS) for each individual at the candidate loci examined (Shankarappa et al., 2022). GRS calculation showed a significantly higher genetic risk score in the overall AUD group (Mean ± SD = 1.4 ± 0.8) compared to the control population group (SAS) (Mean ± SD = 0.82 ± 0.76) (*p* < 0.01). Further, subgroup analysis showed that the AUDC + ve group had a higher GRS (Mean ± SD = 1.3 ± 1.0) than the AUDC-ve group (Mean ± SD = 1.1 ± 1.0) though this was not statistically significant. Thus it appears that effect sizes taken from other studies could successfully predict adverse outcomes of risk allele combinations in our population. However, a more accurate calculation might have to include computed variables to account for cross-population genetic structure. Such a computed GRS could predict the risk of developing cirrhosis on alcohol exposure and have applications in personalised medicine.

### NGS for neuropsychiatry

**FIGURE 2:**
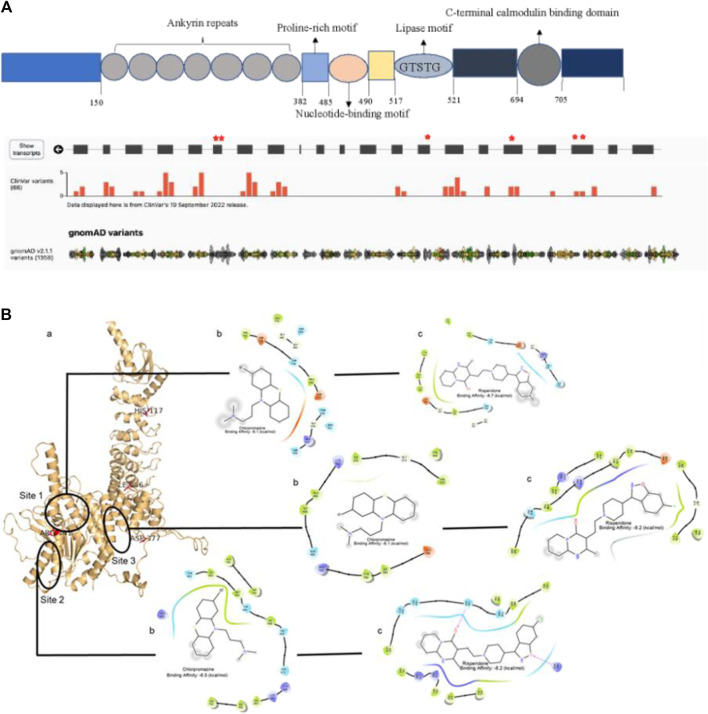
The PLA2G6 protein contains 806 amino acids and harbors various domains including ankyrin repeats, a GXSXG lipase catalytic site, a nucleotide-binding domain, and two binding sites for calmodulin (https://www.uniprot.org/). The gene is highly conserved (dN/dS ratio 2.464; missense variant Z-Score 1.21 (gnomAD)), and is syntenic across many vertebrates. **(A)** The red asterisks denote exons with variants in the current sample. **(B)** Molecular docking analysis of antipsychotics with the predicted structure of PLA2G6 (a) The final full-length structure is shown with the putative binding sites circled in black; site 3 is the interface region between the catalytic domain and the ankyrin repeat domain. The residues that undergo mutation are labelled. Interactions of chlorpromazine (b) and risperidone (c) in the three different binding sites with the PLA2G6 receptor are shown. Hydrophobic interactions (in b,c) are in green, polar interactions in sky blue, negatively and positively charged residue interactions in red and blue respectively. Docking results show that risperidone has a better binding affinity than chlorpromazine. The mutations Arg741--- and Asp377--- are near the binding site 2 and site 3 (interface region between the catalytic domain and the ankyrin repeat) respectively, and thus have a direct or indirect impact on the drug binding, and alter the function of the protein.

We screened our whole exome sequencing data of familial severe mental illness families for *PLA2G6* variants (Figure 2A). WES in 310 individuals from SMI families and population controls, revealed 851 instances (720 intronic, 57 exonic, 56 5′UTR, 18 3′UTR) of variants in the *PLA2G6* gene. Ten exonic variants (4 synonymous and 6 non-synonymous) were seen in 57 individuals. Six non-synonymous variants were found in heterozygous state (Table 2). SIFT and MutationTaster predicted p.H117R, p.I256V and p.D377Y as deleterious. The variant p.Asp377Tyr was found in 3 siblings with schizophrenia, all of whom had pronounced parkinsonian symptoms on antipsychotic treatment. To assess the possible impact of these variants on drug binding, molecular docking analysis with the AlphaFold structure of the protein and antipsychotic medications, chlorpromazine, and risperidone was done. The preliminary molecular docking analysis suggests that while both chlorpromazine and risperidone bind at three predicted drug binding sites, risperidone has a greater binding affinity (Figure 2B). In addition, the variants p.Arg741Gln, p.Arg741Trp (near site 2) and p.Asp377Tyr (near site 3, which is the interface region between the catalytic domain and the ankyrin repeat) are in close proximity to these predicted binding sites. Which could have an impact in the antipsychotic binding affinity of the particular variant protein in addition to alteration in its biological function. It is perhaps not inconceivable that in schizophrenia patients with such heterozygous *PLA2G6* variants, antipsychotic treatment can trigger parkinsonian symptoms.

**TABLE 2:** Clinical and genetic characteristics of pathogenic PLA2G6 variants identified by exome sequencing and pathogenicity predictions obtained from Varsome (D-deleterious, T-tolerated).

No.	GRCh37_Start	Ref	Alt	Locations in ref gene NM_003560	Diagnosis (n)	Pathogenicity prediction			
						SIFT	MutationTaster	FATHMM	MetaSVM
1	chr22:38541520	T	C	exon3:c.A350G:p.H117R	Schizophrenia (1)	D	D	T	T
2	chr22:38541464	G	A	exon3:c.C406T:p.H136Y	Schizophrenia (1)	T	D	T	T
3	chr22:38536020	T	C	exon5:c.A766G:p.I256V	Control (1)	D	D	T	T
4	chr22:38528888	C	T	exon7:c.G1027A:p.A343T	BPAD (4) Controls (4)	T	D	T	T
5	chr22:38525511	G	A	exon8:c.C1136T:p.P379L	Control (1)	T	D	T	T
6	chr22:38525518	C	A	exon8:c.G1129T:p.D377Y	parkinsonism & Schizophrenia (3)	D	D	T	T

## Discussion

A shift from the amyloid hypothesis to genetic causes like the *APOE* Ɛ4 risk haplotype and variants, e.g., *PSEN1* has led to a better understanding of the biology of dementia (van der Ende et al., 2023). After the apolipoprotein locus was consistently reported as a locus of major effect, many GWA studies conducted analysis after masking this region to look for additional risk loci (Reitz et al., 2023). However, the adjoining *TOMM40* region may be equally important in contributing to the risk of Alzheimer’s disease. Our results show that the *TOMM40* locus may have a role in the risk of dementia, even in non *APOE* Ɛ4 carriers. The effect of this locus might be influenced by the genetic structure of the region in different populations. Recent studies provide strong evidence that abnormal cholesterol metabolism by *APOE* Ɛ4 could be linked to AD-associated pathology (Jeong et al., 2019). In an early study we reported, the risk of dementia, both Alzheimer’s dementia (AD) and Vascular dementia (VaD), in those with an *APOE* Ɛ4 haplotype was 3–4 times higher (OR: 3.72 in AD and 2.72 in VaD) (Bharath et al., 2010). Vascular dementia might be the consequence of prolonged hyperlipidemia and impaired cholesterol transport. The lipidation of APOE itself in the brain is driven by the activity of ATP binding cassette proteins ABCA1 and ABCG1 (Lawn et al., 1999). Thus, the prevalence of dementia in different world populations may be influenced by multiple genetic loci, which in turn would be influenced by past population sizes, bottlenecks, admixture and dispersal (Reitz et al., 2023). In addition, environmental factors experienced during a lifetime, like education, nutrition status, lifestyle, *etc.*, Could amplify or attenuate the genetic risks thus making cross population comparisons critical.

Individuals with AUDC + ve started drinking at a later age and were drinking a lesser amount of alcohol compared to the people in AUDC-ve group. We find that GRS of both the groups (AUDC + ve and AUDC-ve) were higher compared to control population. In a similar study, a risk score based on three genetic risk variants and diabetic status enabled the stratification of heavy drinkers based on their risk of cirrhosis, permitting earlier preventive interventions (Whitfield et al., 2022). GRS might be useful to predict the risk of developing cirrhosis before clinical symptoms are felt in individuals who have been drinking heavily.

The environmental influence is exemplified by issues of alcohol use, which differ markedly across populations (Deak et al., 2019). Variation in alcohol metabolizing genes, and its relation to alcohol consumption, dependence, and thus disease, may differ. Evolutionarily, being able to consume alcohol may be as old as the ability of ancient man to consume overripe, fermented fruit which might have fallen from trees. Eventually, a transition from nomadic to agrarian lifestyles led to novel methods of food grain processing and fermentation of cereals in many parts of the world. The invention of distilling at the end of the first millennium, and the use of spirits, perhaps proved lethal for many populations (Butler and Needham, 1980; Ehlers et al., 2004). The climate and lifestyle may have dictated different food and alcohol consumption in different parts of the world. The environment might then play a role in the clinical manifestation of an underlying genetic risk for liver complications.

PLA2G6-related parkinsonism shows a fairly distinct phenotype of young onset parkinsonism/dystonia, gait/balance, and/or psychiatric/cognitive symptoms (Magrinelli et al., 2022). Several population-specific mutations in *PLA2G6* have been reported to cause Infantile neuroaxonal dystrophy and autosomal recessive Parkinsonism (Hanna Al-Shaikh et al., 2022; Wan Y et al., 2022). *PLA2G6* gene-associated neurodegenerative disorders resulting from homozygous c. 2222G > A (p.Arg741Gln) mutation has been described in two cases having variable neuropsychiatric phenotypic and imaging findings from our centre (Sakhardande et al., 2021). While parkinsonism is a well-recognized and common side effect of antipsychotic treatment (Miller, 1997), we speculate that the p.Asp377Tyr variant in the *PLA2G6* gene, which lies close to the antipsychotic binding site (Figure 2B) may influence binding of ligands. Just as other variants in *PLA2G6* have are related to Parkinsonian syndromes, this variant could increase the risk of parkinsonian symptoms in individuals who are exposed to antipsychotics. Since there appears to be a difference in the type and distribution of alleles in the *PLA2G6* gene across populations (Magrinelli et al., 2022), the side effects for particular drugs may also display a population-specific pattern. Evaluating genomes of patients for such variants, might help to predict adverse side effects of certain drugs.

We describe some aspects of our work, to identify loci that may increase genetic susceptibility to disease, and side effects of treatment. Rare variants in particular genes (*PLA2G6*), variations in frequency of common variants (*APOE4 and TOMM40*), and genes that may be stressed by environmental exposures (e.g., to alcohol) all impact risk of neuropsychiatric disease. These highlight that clinical issues may arise by interaction between population-specific genetic variations, and environmental exposures. Variants in the *PLA2G6* gene contribute to an autosomal recessive young onset Parkinsonian syndrome (Park14), Parkinson’s disease, and may even predispose to iatrogenic parkinsonism. These risks may differ across populations, as a consequence of difference in the frequency of the variations that affect the structure and function of PLA2G6 enzyme. The pattern of LD between *APOE Ɛ4* and *TOMM40* differs across populations, and the effect of these may together, or independently contribute to the risk of dementia. Similarly, variations in ADH and ALDH enzymes, in the presence of excessive alcohol use, contribute to different clinical consequences (cirrhosis, cancer, *etc.*). The minor allele frequencies of crucial variants at all the loci discussed here; *PLA2G6, APOE4, TOMM40* and alcohol metabolising enzymes are seen to vary in different populations. Thus the influence of these risk loci in disease prevalence and expression may differ in different world populations.

We acknowledge that our study may have some limitations. It is difficult to comment upon the effect of sex on neuropsychiatric illness. In the present study, the addiction study was carried out on male subjects largely for ease of sampling. There is considerable variation in the sex distribution of dementia cases encountered in the clinic. While we found a larger number of *APOE Ɛ4* carriers in elderly men, this could be the result of a sampling bias. However, there is no difference in the incidence or prevalence between sexes in multiple affected families. In the absence of GWAS data for alcohol liver disease for the SAS population we have used summary statistics from the European PGC study. Further addition of controlling variables for age, alcohol exposure and genetic structure might improve sensitivity and specificity of the computed GRS scores.

Thus to summarise, founder effects, population differences, admixtures as well as protein modelling and cell-models would be necessary to explore the genotype-phenotype issues in detail. Our work on well-characterized clinical samples has provided some useful clues. Genetic loci show variation in effect size which is reflected in a spectrum of phenotypes which may appear unrelated. Genetic information should be studied in the context of ethnicity, clinical phenotype and environment in order to provide a complete picture.

## Data Availability

The raw data supporting the conclusions of this article will be made available by the authors, without undue reservation.

## Supplementary material

Supplementary Figure S1 Relative expression of TOMM40 gene in controls vs AD cases.
