# Supplementary material for: Molecular genetics of neuropsychiatric illness: some musings
Source: Front Genet. 2023 Nov 1;14:1203017. doi: 10.3389/fgene.2023.1203017 (PMC10646253; doi:10.3389/fgene.2023.1203017)
Supplement: Supplementary file 1 [file Table1.docx]

**Supplementary Table 1: Demographic of samples for dementia study**
