# Supplementary figures and images for: Molecular genetics of neuropsychiatric illness: some musings
Source: Front Genet. 2023 Nov 1;14:1203017. doi: 10.3389/fgene.2023.1203017 (PMC10646253; doi:10.3389/fgene.2023.1203017)

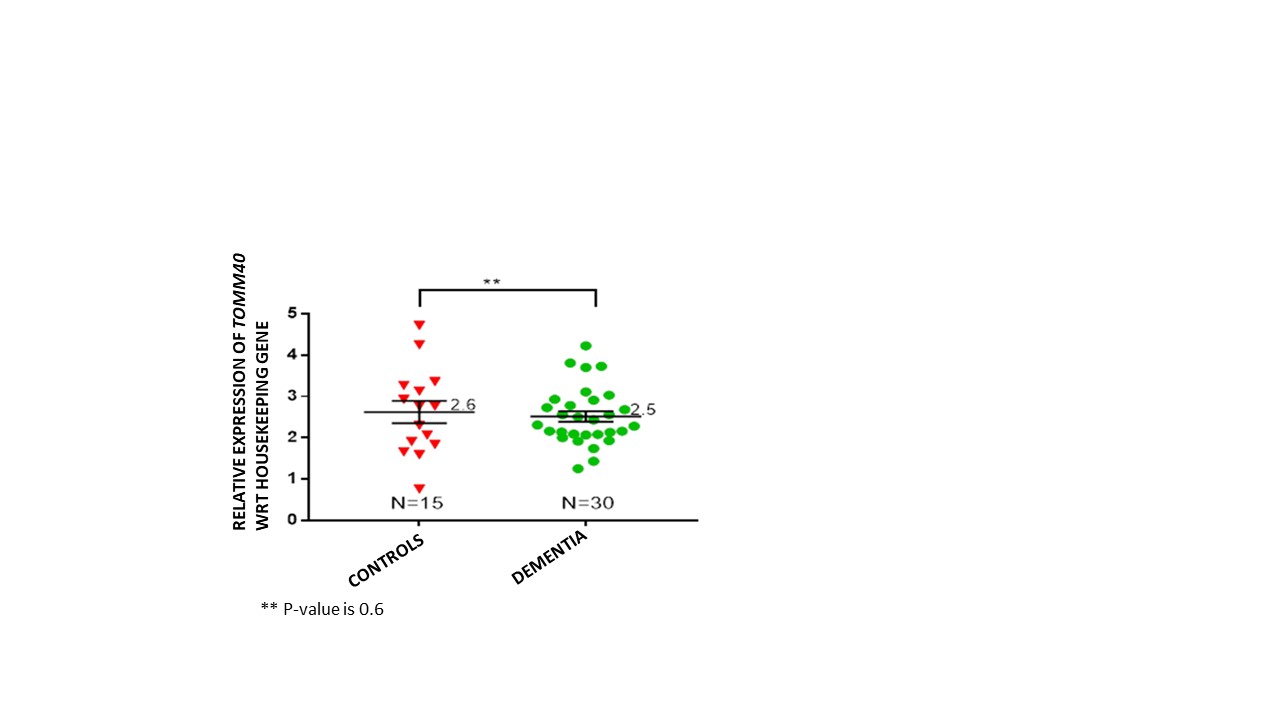

Supplement: Supplementary file 2 [file Image1.jpeg]
