# Supplementary material for: Molecular genetics of neuropsychiatric illness: some musings
Source: Front Genet. 2023 Nov 1;14:1203017. doi: 10.3389/fgene.2023.1203017 (PMC10646253; doi:10.3389/fgene.2023.1203017)
Supplement: Supplementary file 3 [file Table2.docx]

**Supplementary Table 2 : Comparison of sociodemographic and clinical variables in AUDC+ve and AUDC-ve individuals**

| **Study Variable**  **(Mean±SD)**  **Range** | **Whole sample set (N=236)** | | |
| --- | --- | --- | --- |
|  | **AUDC +ve**  **(N=131)** | **AUDC-ve (N=105)** | **P-value** |
| **Age**  **(years)** | 45 ± 9 | 41 ± 10 | 0.01 |
| **Duration of drinking (years)** | 16 ± 7  (1 – 35) | 18 ± 8  (8 – 50) | 0.11 |
| **Amount of Alcohol intake (ml/day)** | 130 ± 68  (20 – 360) | 160 ± 77  (30 – 420) | 0.01 |
| **Age at onset of Drinking (years)** | 29 ± 8  (6 – 51) | 23 ± 7  (18 – 46) | <0.001 |
| **MELD Score** | 23.4 ± 7.5  (9 – 40) | NA | NA |

Footnote: Standard drinks per day (alcohol intake): 1-2 drinks (1 drink = 10 gram absolute alcohol) according to WHO (World Health Organisation) guidelines; MELD (Model for End-Stage Liver Disease)
